# AAV9-Mediated *PTEN* Gene Therapy Rescues Hepatic Pathology and Neuroanatomical Abnormalities in a Murine Model of *PTEN* Hamartoma Tumour Syndrome

**DOI:** 10.3390/pharmaceutics18080922

**Published:** 2026-07-27

**Authors:** Ceren Erdem, Sophie Thomson, Noha Bahey, Jim Selfridge, Timothy Kendall, Stuart Cobb, Kamal Gadalla

**Affiliations:** 1 Simons Initiative for the Developing Brain, Centre for Discovery Brain Sciences, University of Edinburgh, Edinburgh EH8 9XD, UK; cerdem@pau.edu.tr (C.E.); sthomso6@staffmail.ed.ac.uk (S.T.); nohabahey@gmail.com (N.B.); jim.selfridge@ed.ac.uk (J.S.); stuart.cobb@ed.ac.uk (S.C.); 2 Department of Medical Biology, Faculty of Medicine, Pamukkale University, Denizli 20070, Türkiye; 3 Department of Histology, Faculty of Medicine, Tanta University, Tanta 31527, Egypt; 4 Centre for Inflammation Research, Institute for Regeneration and Repair, University of Edinburgh, Edinburgh EH16 4UU, UK; tim.kendall@ed.ac.uk; 5 Centre for Systems Health and Integrated Metabolic Research (SHiMR), Department of Biosciences, School of Science and Technology, Nottingham Trent University, Nottingham NG11 8NS, UK

**Keywords:** *PTEN* Hamartoma Tumour Syndrome, heterozygous *Pten* mouse model, gene therapy, adeno-associated virus (AAV9)

## Abstract

**Background/Objectives**: *PTEN* Hamartoma Tumour Syndrome (PHTS) is a rare inherited disorder caused by germline *PTEN* mutations, presenting with cancer predisposition and neurodevelopmental abnormalities, including macrocephaly. PTEN functions as a tumour suppressor by regulating the PI3K/AKT/mTOR pathway and contributes to cellular adhesion, migration, and genomic stability. As no curative therapy exists, gene therapy represents a promising avenue to correct the underlying genetic defect. **Methods**: In this study we evaluated the therapeutic potential of AAV9-mediated *PTEN* gene delivery in a *Pten*^Δ5/+^ mouse model, which is predominantly characterized by progressive lymphoid hyperplasia leading to lymph node tumour development, as well as hepatic focal lesions and macrocephaly. **Results**: Systemic vector delivery did not improve lymph node enlargement which represents the predominant disease phenotype in the *Pten*^Δ5/+^ mice at the doses tested, due to restricted vector biodistribution, but it did effectively rescue the hepatic lesions, including steatosis and steatohepatitis-like changes. Mechanistically, AAV-mediated PTEN expression significantly reduced elevated p-AKT levels, indicating attenuated hyperactivation of the PI3K/AKT/mTOR pathway. Additionally, intracranial delivery of the same vector in *Pten*^Δ5/+^ mouse neonates ameliorated macrocephaly without apparent adverse effects. **Conclusions**: These findings highlight the therapeutic potential of AAV9-mediated *PTEN* gene therapy in PHTS, particularly for hepatic and neuroanatomical manifestations. Optimization of vector biodistribution and transduction efficiency will be critical for clinical translation.

## 1. Introduction

*PTEN* Hamartoma Tumour Syndrome (PHTS; OMIM 158350) encompasses a heterogeneous group of disorders arising from germline mutations in the *PTEN* gene (OMIM 601728), which are inherited in an autosomal dominant manner [1,2]. The phenotypic spectrum includes several overlapping inherited cancer predisposition syndromes—such as Cowden syndrome, Lhermitte–Duclos disease, Bannayan–Riley–Ruvalcaba syndrome, and Proteus and Proteus-like syndromes—reflecting the pleiotropic impact of PTEN dysfunction [3,4,5].

In addition to oncogenic risk, *PTEN* mutations contribute to the pathogenesis of neurodevelopmental disorders, notably autism spectrum disorder (ASD) in the context of macrocephaly [6]. Individuals with PHTS frequently exhibit dermatological manifestations (trichilemmomas, lipomas, fibromas, papillomas), gastrointestinal polyposis, increased carcinoma risk, and neurological involvement, including macrocephaly, seizures, developmental delay, and ASD [7,8,9].

PTEN functions as a canonical tumour suppressor [10] and is among the most frequently mutated tumour suppressors in sporadic human cancers [11,12,13]. Most of the *PTEN* germline mutations cluster within exons 5, 7, and 8, with exon 5 (encoding the catalytic phosphatase core) accounting for 40% of cases [14,15]. Functional assessment using murine models demonstrates that homozygous *Pten* knockout results in early embryonic lethality, whereas heterozygous deletion, particularly including exon 5, recapitulates PHTS pathology [16,17,18]. The onset and severity of tumour development are significantly modulated by genetic background, with phenotypic variability observed even among models carrying the same mutation [16,18,19,20].

At present, no targeted therapies exist for PHTS, and clinical management is limited to surveillance and treatment of neoplastic and non-neoplastic manifestations. Pharmacological inhibition of the PI3K/AKT/mTOR pathway, while mechanistically rational due to its negative regulation by PTEN, has exhibited limited clinical benefit in patients with germline *PTEN* mutations [21,22,23]. PTEN’s tumour suppressive activity is not restricted to its lipid phosphatase function; the protein also displays protein phosphatase activity and scaffolding roles within both cytoplasmic and nuclear compartments [24,25]. Therefore, comprehensive restoration of PTEN activity has the potential to address both canonical and non-canonical disease mechanisms in PHTS.

In the absence of disease-modifying therapies, lifelong surveillance remains essential for PHTS patients to enable early cancer detection. The ubiquitous expression and small coding size of *PTEN*, which facilitates efficient vector incorporation, support the development of gene therapy approaches aimed at correcting the underlying molecular defect. In addition, previous studies have reported extracellular vesicle-mediated transfer of canonical PTEN, suggesting the potential for intercellular dissemination of PTEN activity [26,27]. Therefore, the objective of this study was to assess the therapeutic impact of AAV9-mediated *PTEN* delivery on the peripheral manifestations, including lymph node enlargement and hepatic pathology, and the neuroanatomical abnormalities characteristic of the *Pten*^Δ5/+^ mouse model, using systemic and intracranial routes of administration, respectively. Following systemic administration, the treatment effectively rescued hepatic lesions but did not reduce lymph node tumour burden, likely due to limited transgene expression in these tissues. In contrast, intracerebroventricular delivery significantly reduced macrocephaly, and importantly, PTEN overexpression in the brain did not produce any detectable adverse effects under the experimental conditions used, supporting a potential safety profile for this approach.

## 2. Materials and Methods

**Animals**: The PTEN heterozygous mouse model (*Pten*^Δ5/+^) was generated on a C57BL/6.

NCrl background by in vitro fertilization of wild-type C57BL/6 oocytes (Central Transgenic Core, the University of Edinburgh) with sperm from males homozygous for the floxed exon 5 allele of *Pten* (*Pten^floxed exon5/floxed exon 5^*), both obtained from the Central Transgenic Core, the University of Edinburgh. Following fertilization, the zygotes underwent Cre recombinase-mediated excision of exon 5 at the morula stage. The edited embryos were subsequently implanted into wild-type C57BL/6 female recipients. Female offspring carrying the *Pten*^Δ5/+^ genotype were then bred with wild-type males to establish the experimental colony. Experimental mice were randomly assigned to treatment groups and housed in groups of 2–5, with different treatment groups represented within each cage so that the individual mouse served as the experimental unit. Investigators responsible for data collection and analysis were blinded to group allocation. *Pten*^Δ5/+^ mice were monitored weekly for tumour development. All palpable tumours were measured using digital callipers, and tumour volumes were calculated according to the formula Volume = (Length × Width^2^)/2. For mice bearing multiple tumours, total tumour burden was calculated as the cumulative volume of all tumours present at each assessment.

All experiments were conducted in accordance with the local ethical approval and under the UK Animals (Scientific Procedures) Act (1986). All mice were genotyped as described previously [28]. Briefly, genomic DNA was isolated from ear biopsies using DirectPCR^®^ Lysis Reagent. To identify *Pten*^Δ5/+^ mice, the wild-type allele was amplified using primers PTENp1 (5′-ACTCAAGGCAGGGATGAGC-3′) and PTENp3 (5′-AATCTAGGGCCTCTTGTGCC-3′), while the mutant allele was detected using PTENp1 and PTENp4 (5′-GCTTGATATCGAATTCCTGCAGC-3′). PCR amplification was performed using DreamTaq Green PCR Master Mix (2×) (Thermo Scientific™, Thermo Fisher Scientific, Waltham, MA, USA; Cat. No. K1082) under the following conditions: initial denaturation at 95 °C for 3 min; 35 cycles of denaturation at 95 °C for 30 s, annealing at 58 °C for 30 s, and extension at 72 °C for 1 min; followed by a final extension at 72 °C for 10 min.

**Vector design and development**: In this study, a scAAV9 vector carrying the Cβh-codon-optimized human PTEN (Cβh-co-*hPTEN*) therapeutic cassette was used. The most abundant isoform, PTEN transcript variant 1 (NM_000314.8), was codon-optimized using the Integrated DNA Technologies IDT Codon Optimization Tool. To ensure strong and widespread expression in peripheral tissues and the CNS, the Cβh promoter—comprising a CMV enhancer, chicken β-actin (CβA) promoter, and a hybrid intron from chicken β-actin and CMV VP sequences [29]—was selected. A Kozak sequence was introduced upstream of the ATG start codon to enhance translation, and a synthetic poly(A) signal was added downstream. The entire construct, flanked by AAV2 ITRs, was synthesized by GeneArt (Thermo Fisher Scientific, Waltham, MA, USA) and cloned into an AAV9 vector. Plasmids were amplified in NEB Stable Competent *E. coli* cells (C3040H; New England Biolabs, Ipswich, MA, USA) and purified using the QIAGEN Maxi Prep Kit (cat. no. 12963, QIAGEN, Hilden, Germany) according to the manufacturer’s instructions. The integrity of the Maxiprep plasmids was verified by MiSeq sequencing (Illumina, San Diego, CA, USA). Virus particles were produced and purified by the Viral Vector Core Facility (UPV) at the Universitat Autònoma de Barcelona. Vector titres were determined as viral genomes per millilitre (vg/mL) by quantification of encapsidated vector genomes using the PicoGreen fluorescence assay, with quantitative PCR (qPCR) used for titre confirmation. Final vector preparations passed routine sterility and mycoplasma quality control testing prior to experimental use. Additional quality attributes, including empty/full capsid ratio and endotoxin levels, were not available from the vector production facility and were therefore not assessed in the present study.

**Injection**: For systemic administration, 100 µL of the scAAV9/Cβh-*hPTEN* viral vector at specified doses (5E11 or 2E12 vg/mouse) was delivered via lateral tail vein injection in female mice. Also, sterile-filtered phosphate-buffered saline (PBS) was used as a vehicle control. To promote vein dilation, mice were pre-warmed in a 37 °C heating chamber for 10–15 min. Following disinfecting the tail with 70% ethanol, mice were placed in a mechanical restraint device (to ensure stability) and injections were performed using a BD Microfine+ Demi U100 0.3 mL syringe (Becton, Dickinson and Company, Franklin Lakes, NJ, USA) over 20 s duration. The injection site was compressed for 1 min to prevent bleeding and virus backflow.

For intracerebroventricular (ICV) injection, neonatal female pups were genotyped and injected with the viral vector at postnatal day 1 (P1). Pups were anesthetized with isoflurane during the procedure. The scalp was disinfected with an iodine solution and dried with a sterile cotton swab. 5E10 or 1E11 vg/mouse in a final volume of 10 µL (5 µL injection per site) was administered bilaterally using a 30G needle attached to a 50 µL Hamilton syringe (Hamilton Company, Reno, NV, USA). Injections were made 1 mm lateral to the midline and anterior to lambda, to a depth of 3 mm, maintained using a custom needle guard. The vector was delivered over 30 s, and the needle remained in place for ~ 50 s post-injection to prevent backflow. Following the procedure, pups were allowed to recover before being returned to their home cage, where they were gently stroked with bedding material to minimize maternal rejection.

**Inclusion and exclusion criteria**

For the IV studies, female *Pten*^Δ5/+^ and wild-type mice aged 5–6 weeks and weighing 15–20 g were included. Mice weighing <15 g or >20 g and animals with unsuccessful tail vein injections were excluded. Successful IV administration was defined as delivery of the full injection volume via the tail vein without evidence of extravasation. For the ICV studies, *Pten*^Δ5/+^ and wild-type neonatal mice were injected at P1, and older pups were excluded. Successful ICV administration was defined by completion of the injection procedure using the established protocol and anatomical coordinates. Only animals that met the inclusion criteria and successfully received the assigned treatment were included in the final analysis. No animals were excluded after successful treatment administration and no post hoc exclusions were applied.

**Behavioural Tests**:

Social approach test: Social approach was assessed using the three-chamber test. The apparatus consisted of a rectangular plexiglass box divided into three equal chambers (20 × 40 × 22 cm), with doors allowing movement between them. After a 20 min acclimation period in the testing room (under white light), mice were allowed to explore the empty apparatus for 5 min. For the test, an unfamiliar, age- and sex-matched mouse was placed under a wire cup in the left chamber, while an empty wire cup (novel object) was placed in the right chamber. Test mice were introduced into the central chamber, and their behaviour was recorded for 10 min using an overhead digital camera (Basler AG, Ahrensburg, Germany; cat. 104845-10). Time spent in each chamber and proximity to the wire cups (sniffing time) was automatically tracked and analyzed using EthoVision XT v12 software (Noldus Information Technology, Wageningen, The Netherlands).

**Vector biodistribution**: To assess the biodistribution of the scAAV9/Cβh-*hPTEN* vector, genomic DNA was extracted using the DNeasy^®^ Blood & Tissue Kit (QIAGEN, Hilden, Germany; Cat #69504) and primers targeting the human PTEN gene (*hPTEN*_Forward: 5′-ATCACGTCGCCGCTATTC-3′; *hPTEN*_Reverse: 5′-ACATATCGCCGTTGAGAAGG-3′) were designed using SnapGene^®^ software, version 7.2 (Dotmatics, Boston, MA, USA) and validated for specificity against the mouse genome using Primer-BLAST. Mouse Actin primers (mActin_Forward: 5′-AGCCATGTACGTAGCCATCC-3′; mActin_Reverse: 5′-CTCTCAGCTGTGGTGGTGAA-3′) were used for normalization.

Quantitative PCR (qPCR) was performed using the StepOnePlus™ Real-Time PCR System (Applied Biosystems, Waltham, MA, USA) and PerfeCTa SYBR^®^ Green FastMix ROX (Quantabio, Beverly, MA, USA). Absolute quantification was performed using a standard curve generated from serial dilutions of synthetic PTEN gBlock DNA of known copy number. Vector genome copy numbers in unknown samples were interpolated from the standard curve and normalized to the genomic copy number of the endogenous Actb gene, which was determined using an Actb gBlock DNA standard of known copy number. The measured Actb copy number was divided by two to estimate the number of diploid mouse genomes, and vector biodistribution was expressed as vector genomes (vg) per diploid genome.

**Western blot**: Total protein was extracted from snap-frozen tissues using a Bead Mill 24 homogenizer (ThermoFisher Scientific, Waltham, MA, USA) in RIPA buffer (10 mM Tris-HCl pH 8.0, 140 mM NaCl, 1% Triton X-100, 0.1% SDS, 0.1% sodium deoxycholate, 1 mM EDTA) supplemented with protease and phosphatase inhibitors. For 4% PFA-fixed brain tissues, 50 µm sections were obtained using a vibratome (Leica VT1000 S; Leica Microsystems, Wetzlar, Germany) and cerebral regions were dissected for protein extraction following the protocol by Thacker et al. [30] using a modified RIPA buffer. Protein lysates were denatured in 1× Laemmli buffer at 95 °C for 10 min. Equal protein amounts and molecular weight markers (Bio-Rad Laboratories, Hecules, CA, USA) were loaded and separated via SDS-PAGE (1× Tris/Glycine/SDS buffer), run at 50 V for 30 min followed by 150 V for 1 h. Proteins were transferred to 0.45 µm nitrocellulose membranes using 1× transfer buffer (with 20% methanol) at 85 V for 2 h. Membranes were rinsed, stained with Revert 700 Total Protein Stain (Li-Cor), and imaged using an Odyssey scanner (Li-Cor) at low intensity. After blocking with Intercept^®^ (TBS) buffer (Li-Cor Biosciences, Lincoln, NE, USA) for 1 h, membranes were incubated overnight at 4 °C with primary antibodies (1:1000) against PTEN (#9559), p-AKT Ser473 (#9271), and pan-AKT (#4691) (Cell Signalling Technology, Danvers, MA, USA). Following washes with TBS-T (0.1% Tween-20), membranes were incubated with appropriate IRDye^®^ secondary antibodies (Li-Cor Biosciences, Lincoln, NE, USA; 1:10,000 dilution). Imaging and quantification were performed with Image Studio v5.2 software (LI-COR Biosciences, Lincoln, NE, USA). Total protein staining served as the loading control, and background correction was applied using top and bottom average signals. Relative protein expression levels were normalized to control or wild-type groups.

**Histological staining**:

**Haematoxylin and eosin (H&E) staining**: Following post-fixation in 4% paraformaldehyde (PFA) in 0.1 M phosphate buffer (PB; Sigma-Aldrich, St. Louis, MO, USA; cat. 158127), tissues were washed twice in 0.1 M PB and subsequently dehydrated through a graded ethanol serial (70% to 100%) before paraffin embedding. Formalin-fixed paraffin-embedded (FFPE) samples were sectioned at 7 µm using a microtome (Leica HistoCore MULTICUT; Leica Biosystems, Nussloch, Germany) and mounted on Superfrost Plus slides (Thermo Scientific™, Thermo Fisher Scientific, Waltham, MA, USA). Slides were dried overnight in a 37 °C incubator. Slides were then dewaxed in xylene (20 min + 5 min fresh), rehydrated through descending ethanol series (100%, 90%, 70%; 30 s each), and rinsed in running water. Nuclei were stained with Harris’ hematoxylin for 3 min, followed by rinsing, brief differentiation in acid alcohol (70% ethanol with 1% HCl), and bluing in Scott’s tap water substitute (20 g MgSO_4_ and 3.5 g NaHCO_3_ in 1 L distilled water) for 3 min. Cytoplasm and extracellular matrix were stained with 1% aqueous eosin for 2 min, followed by a brief rinse and treatment with 5% potassium alum.

Liver section slides were dehydrated through ascending ethanol series (70%, 90%, 95%, 2 × 100%), cleared in xylene, and mounted with DPX. Whole-slide imaging was performed using a NanoZoomer brightfield slide scanner (Hamamatsu Photonics K.K., Hamamatsu City, Shizuoka, Japan), and images were analyzed with NDP.view2 software, version 2.9.29 (Hamamatsu Photonics K.K., Hamamatsu City, Shizuoka, Japan).

Histological evaluation of H&E-stained liver sections was performed independently by two investigators who were blinded to the treatment groups. The histopathological findings were subsequently reviewed and confirmed by an experienced liver pathologist.

**Immunohistochemistry (IHC):** Unless stated otherwise, IHC was performed on formalin-fixed paraffin-embedded (FFPE) tissue sections. After dewaxing, heat-induced antigen retrieval was conducted in 10 mM sodium citrate buffer (Sigma-Aldrich, St. Louis, MO, USA; cat. C3674) containing 0.05% Tween-20 (BioRad, cat. 1706531) at 85 °C for 30 min in a pre-warmed water bath. Slides were cooled to room temperature in the same buffer and then washed twice (5 min each) in 0.3 M PBST (0.3 M NaCl, 10% 0.2 M phosphate buffer, 0.3% Triton X-100). Staining was performed using rabbit- or mouse-specific HRP/DAB (ABC) kits (Abcam, Cambridge, UK; cat. ab64261 or ab64259), depending on the primary antibody. Sections were incubated overnight at 4 °C with primary antibodies diluted in 0.3 M PBST containing 5% normal goat serum (Sigma, cat. G9023): anti-PTEN (1:500, Cell Signalling #9559), or anti-FLAG (1:400, Millipore F1804). The following day, slides were washed and incubated sequentially with biotinylated goat anti-polyvalent reagent (10 min) and streptavidin–HRP (10 min), followed by three 10 min washes with PBST. DAB chromogen (Vector Laboratories, Newark, CA, USA; cat. SK-4100) was applied for 10 min, and counterstaining was performed with Haematoxylin Grill No:1 (Sigma-Aldrich, St. Louis, MO, USA; cat. GH132) for 30 s. Slides were rinsed in tap water for 3 min, dehydrated through graded ethanol (70%, 90%, 95%, and 100%; 30 s each), cleared in xylene, and mounted with DPX (Sigma-Aldrich). Slides were air-dried prior to imaging.

For corpus callosum analysis, 50 µm-thick fixed section from the right hemisphere (n = 5 mice per group; 4 sections per mouse) were prepared and stained with NeuroTrace™ 500/525 Green Fluorescent Nissl Stain (Invitrogen^TM^, Thermo Fisher Scientific, Waltham, MA, USA; cat. N21479) and Hoechst 33342 (Thermo Scientific™, Thermo Fisher Scientific, Waltham, MA, USA; cat. 62249). Images were acquired using a Nikon A1R confocal microscope and these images were analyzed with Leica LAS X software, version 4.1 (Leica Microsystems GmbH, Wetzlar, Germany).

**Isotropic Fractionation**: Cell nuclei were quantified using the isotropic fractionator method involving chemical and mechanical tissue dissociation [31,32]. Briefly, after transcardial perfusion with 4% PFA and post-fixation for 2–3 weeks, the left hemisphere was dissected to isolate the cerebral cortex, cerebellum, olfactory bulb, and remaining structures. The cerebral cortex was homogenized in 40 mM sodium citrate, 1% Triton X-100 using a 15 mL glass tissue grinder (Kimble™, DWK Life Sciences, Millville, NJ, USA) for up to 30 min. The nuclear suspension was centrifuged at 4000× *g* for 10 min at 4 °C, and the pellet was resuspended in 10 mL of 0.1M PBS with Hoechst 33342 (2 µg/mL) (Thermo Scientific™, Thermo Fisher Scientific, Waltham, MA, USA; cat. 62249).

Four 20 µL aliquots were loaded into an improved Neubauer chamber and nuclei were counted under fluorescence microscopy (Olympus Corporation, Tokyo, Japan) within the central 1 mm^2^ grid (0.0001 mL) (at least 50 nuclei) four times for each mouse. Counts were averaged across replicates; additional counts were performed if the coefficient of variation (CV) exceeded 0.15. Final estimates were calculated by multiplying the average count by 10.

**Statistical Analysis**: All analyses were performed using GraphPad Prism v9. Statistical tests were chosen based on data distribution and test`s assumptions. Data normality was assessed using the Shapiro–Wilk test before selecting parametric analyses. Parametric tests (two-tailed Student’s *t*-test, one-way or two-way ANOVA, including repeated measures) were used when normality was assumed; Welch’s correction was applied when variances were unequal. For non-parametric data, two tailed Mann–Whitney, Kruskal–Wallis, or Log-rank tests were applied. Post hoc multiple comparison tests were conducted where appropriate and are detailed in the text. A *p*-value < 0.05 was considered statistically significant.

## 3. Results

### 3.1. Pten^Δ5/+^ Female Mice Developed Early, Aggressive Tumours, Making Them an Optimal Model for Gene Therapy Evaluation

To validate the phenotype of our *Pten*^Δ5/+^ mouse colony, we first characterized the genotype–phenotype relationship associated with the exon 5 deletion by comparing *Pten*^Δ5/+^ mice with their wild-type littermates, as previous studies [16,18,20] have reported phenotypic heterogeneity in mouse models of *Pten* haploinsufficiency. *Pten*^Δ5/+^ female mice showed significantly earlier tumour onset than males and by week 36, all female *Pten*^Δ5/+^ mice developed tumours, compared to only 25% of males (Figure S1A). Additionally, total tumour growth rates, determined by weekly digital calliper measurements and calculated as the cumulative volume of all tumours present in each mouse, were also higher in *Pten*^Δ5/+^ females than in male mice (Figure S1B,C). Despite prior reports of variable behavioural phenotypes, comprehensive testing revealed no deficits in locomotor activity, anxiety-like behaviour, or sociability in *Pten*^Δ5/+^ mice compared to wild-type controls (Figure S1D–K). However, postmortem brain analysis showed that both *Pten*^Δ5/+^ females and males had significantly bigger brain mass (593.1 ± 25.9 mg and 563.6 ± 27.5 mg, respectively) compared to the corresponding wild-type controls (females: 473.8 ± 13.9 mg; males: 455.5 ± 13.7 mg) (Figure S1L–N).

### 3.2. Systemic scAAV9/Cβh-hPTEN Fails to Ameliorate Lymph Node Neoplasia in Pten^Δ5/+^Female Mice

**Figure 1:**
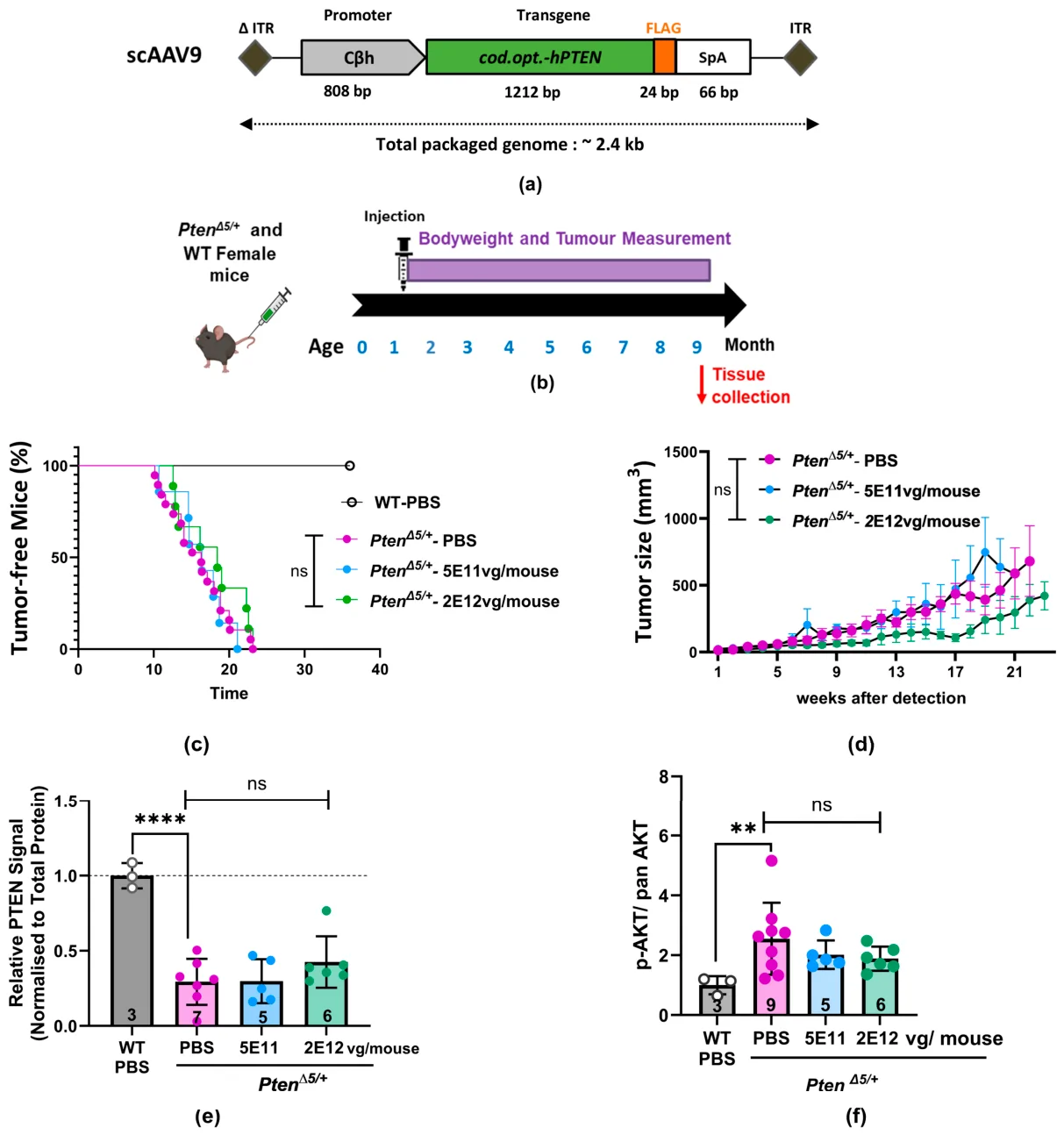
Systemic delivery of scAAV9/Cβh-*hPTEN* in *Pten*^Δ5/+^ female mice has no significant effect on lymph nodes neoplasia: (**a**) Schematic diagram of scAAV9/Cβh-*hPTEN* viral vector. (**b**) Experimental plan: *Pten*^Δ5/+^ females received tail vein injections of either PBS or scAAV9/Cβh-*hPTEN* (5E11 or 2E12 vg/mouse) at 5 weeks of age; wild-type females received PBS as a control. Mice were monitored weekly for body weight and tumour development until the age of 36 weeks. (**c**) Kaplan–Meier curves showing tumour onset in *Pten*^Δ5/+^ females across treatment groups (Log-rank (Mantel–Cox) test; *Pten*^Δ5/+^ PBS *n* = 17; 5E11 vg/mouse *n* = 7; 2E12 vg/mouse *n* = 9; ns = not significant). (**d**) In-life weekly tumour growth measured from first detection to study endpoint (mixed-effects model, type III). (**e**) Quantification of PTEN protein levels via Western blot (Student’s *t*-test for wild-type PBS (*n* = 3) vs. *Pten*^Δ5/+^ PBS (*n* = 7); one-way ANOVA for treated groups; *Pten*^Δ5/+^ PBS *n* = 7; 5E11vg/mouse *n* = 5; 2E12 vg/mouse *n* = 6). (**f**) Quantification of relative p-AKT(Ser473) levels normalized to pan-AKT (Student’s *t*-test for wild-type PBS vs. *Pten*^Δ5/+^ PBS; Welch’s ANOVA for treated groups). Data are presented as mean ± SD with individual data points shown (circles). ** *p* < 0.001, **** *p* < 0.0001; ns = not significant.

To test the therapeutic potential of gene therapy in PTEN haploinsufficiency, we developed a self-complementary AAV9 vector expressing codon-optimized, C-terminally Flag-tagged human *PTEN* (canonical isoform-coding sequence, NM_000314.8) under control of the chicken β-actin promoter with CMV enhancer (Cβh) (Figure 1a). Five-week-old female *Pten*^Δ5/+^ mice received a tail vein injection of either scAAV9/Cβh-*hPTEN* at 5E11 vg/mouse (2.5E13 vg/kg-low dose), 2E12 vg/mouse (1E14 vg/kg-high dose) or phosphate-buffered saline (PBS) as a vehicle control. Wild-type female littermates received an IV PBS injection as a vehicle-treated control. The incidence and size of palpable subcutaneous tumours, and body weight were tracked weekly (Figure 1b) over a nine-month period using a blinded assessment strategy.

No significant difference in median tumour onset time—as detected by routine inspection and palpation—was observed between the vector-treated groups (5E11: 16.4 weeks, 2E12: 18.4 weeks) and PBS controls (16.3 weeks, Figure 1c). Growth rates of individual tumours were similar between PBS vehicle control and vector treated groups (Figure 1d). Postmortem histopathological analysis revealed that the majority of tumours originated as neoplastic lesions within the cervical and inguinal lymph nodes (Figure S2A), establishing lymph node neoplasia as the principal disease burden in this model.

To elucidate the lack of therapeutic effect at the doses tested, we assessed vector biodistribution and PTEN expression in the lymph nodes across the experimental groups. Vector genome biodistribution analysis indicated minimal accumulation of scAAV9/Cβh-*hPTEN* vector (Figure S2B). Consistently, Western blot analysis showed that PTEN levels in the lymph nodes of vector-treated *Pten*^Δ5/+^ mice remained significantly reduced—approximately 70% (5E11 vg/mouse) and 60% (2E12 vg/mouse) of wild-type levels—and were not statistically different from PBS-treated *Pten*^Δ5/+^ controls (Figure 1e and Figure S2C). Additionally, phosphorylated AKT (p-AKT) and pan-AKT levels, which were elevated in *Pten*^Δ5/+^ lymph nodes, were not significantly attenuated by the gene therapy (Figure 1f and Figure S2C).

### 3.3. Systemic Delivery of scAAV9/Cβh-hPTEN Significantly Reduces Hepatic Focal Lesions in Pten^Δ5/+^ Female Mice

**Figure 2:**
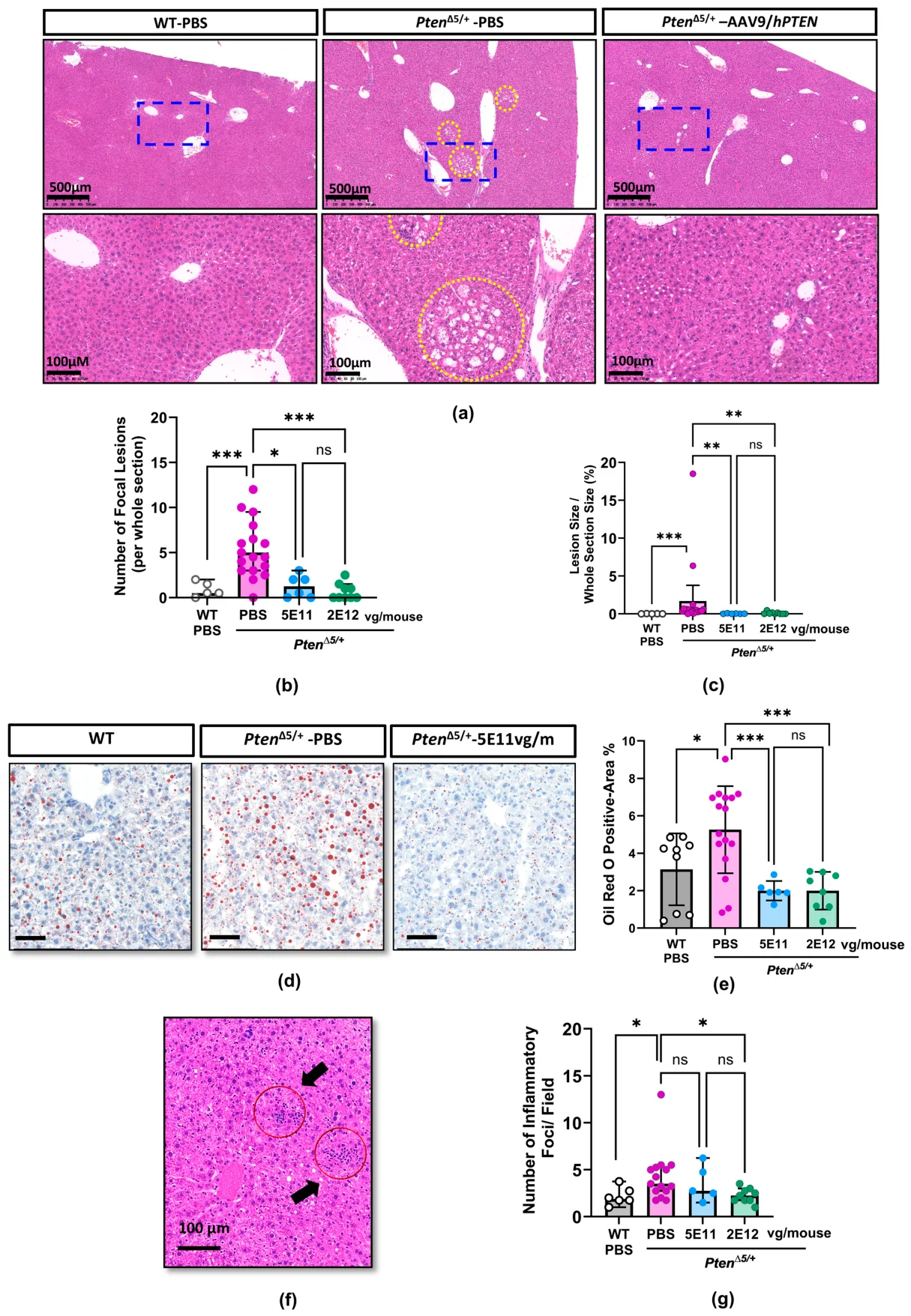
Systemic treatment of scAAV9/Cβh-*hPTEN* significantly reduced hepatic focal lesions and inflammatory burden in the liver of *Pten*^Δ5/+^ mice: (**a**) Representative H&E-stained liver sections from different treatment groups. Yellow circles indicate focal hepatic lesions in *Pten*^Δ5/+^ mice; dashed blue boxes show areas magnified below. (**b**) Quantification of focal hepatic lesions per section. Lesion counts were significantly elevated in *Pten*^Δ5/+^ PBS mice compared to wild-type (WT) controls (Mann–Whitney test; U = 5, *p* = 0.0008; median (95% CI)), but were reduced following vector treatment (Kruskal–Wallis with Dunn’s test; H = 19.53; Median (95% CI) *p* < 0.0001; *Pten*^Δ5/+^ PBS *n* = 17; 5E11 vg/mouse *n* = 6; 2E12 vg/mouse *n* = 9). (**c**) Total lesion area as a percentage of the whole liver section. Lesion burden was significantly higher in *Pten*^Δ5/+^ PBS mice versus wild-type (two-tailed Mann–Whitney test; U = 4, *p* = 0.0006; median (95% CI) and significantly reduced with scAAV9/Cβ*h-hPTEN* treatment (Kruskal–Wallis; H = 17.07, *p* = 0.0002; median (95% CI)). (**d**) Representative images of liver sections stained with Oil Red O (scale bar: 50 µm). (**e**) Graph showing quantitative analysis of ORO-stained liver sections among the treatment groups wild-type PBS (*n* = 9), *Pten*^Δ5/+^ PBS (*n* = 16) and *Pten*^Δ5/+^ 5E11 vg/mouse (*n* = 6) and 2E12 vg/ mouse (*n* = 8) (two-tailed Student’s *t*-test with Welch’s correction for genotype differences *p* = 0.0239 and Welch’s ANOVA with Dunnett’s T3 multiple comparisons test for treatment comparison, *p* = 0.0003). (**f**) Representative image of H&E-stained liver section of PBS-treated *Pten*^Δ5/+^ mice showing inflammation foci (red circles and arrows). (**g**) Quantification of inflammatory foci per section. Points, represented as circles within each bar, correspond to individual mice (Kruskal–Wallis; H = 6.218, *p* = 0.0446; median (95% CI)). Bar graphs display mean ± SD unless otherwise indicated. Significance levels: * *p* < 0.05, ** *p* < 0.01, *** *p* < 0.001; ns = not significant.

Gross postmortem examination revealed liver abnormalities, including gross enlargement and white macroscopic foci, in 61% of PBS-treated *Pten*^Δ5/+^ mice, absent in wild-type controls and markedly diminished in scAAV9/Cβh-*hPTEN*-treated groups. Histopathological evaluation with H&E staining confirmed focal hepatic fatty lesions characterized by circular lipid droplets and increased mononuclear inflammatory infiltrates in untreated *Pten*^Δ5/+^ mice. Quantitative analysis demonstrated a significant increase in the number (*p* = 0.0008) and overall area (*p* = 0.0006) of focal lesions in PBS-treated *Pten*^Δ5/+^ livers compared to wild-type controls, both of which were significantly reduced following scAAV9/Cβh-*hPTEN* administration at the doses tested (Figure 2a–c). Lipid-specific Oil Red O staining confirmed accumulation of hepatic fat vesicles in PBS-treated *Pten*^Δ5/+^ mice (Figure 2d), and quantification of Oil Red O-positive staining showed significant reduction in lipid deposition in vector-treated *Pten*^Δ5/+^ mice (Figure 2e), which corroborates histopathological findings and emphasizes the therapeutic efficacy of scAAV9/Cβh-*hPTEN* in mitigating hepatic steatosis. Additionally, the incidence of inflammatory foci, defined as discrete regions containing ≥ 10 inflammatory cells, was significantly higher in PBS-treated *Pten*^Δ5/+^ mice than in wild-type controls (*p* = 0.02). Administration of the higher AAV9-PTEN dose (2E12 vg/mouse) significantly reduced inflammatory foci incidence compared with PBS-treated *Pten*^Δ5/+^ mice (*p* = 0.04) (Figure 2f,g).

**Figure 3:**
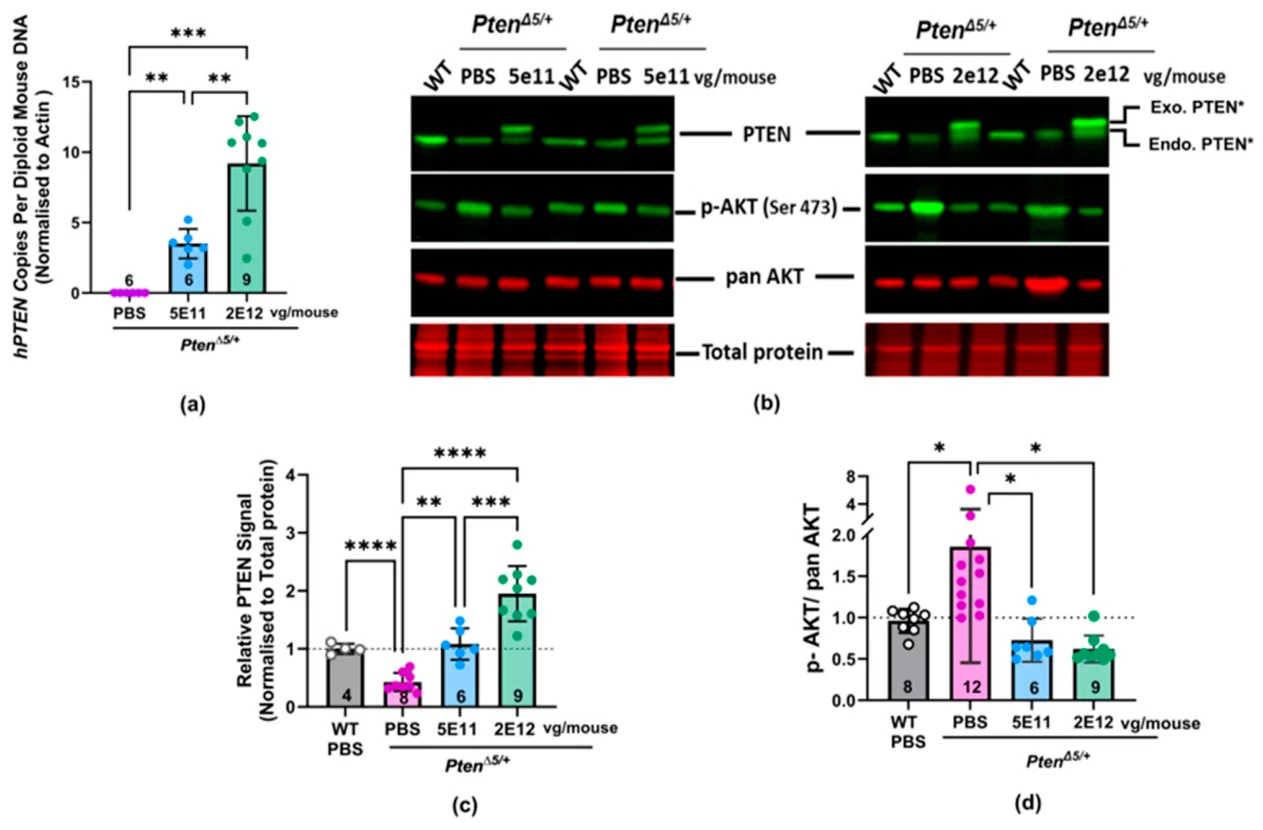
Systemic treatment of scAAV9/Cβh-*hPTEN* restored PTEN levels and normalized p-AKT/pan-AKT signalling in the liver of *Pten*^Δ5/+^ mice: (**a**) Biodistribution of scAAV9/Cβh-*hPTEN* vector in liver tissue 8 months post-injection, measured by qPCR (Welch’s ANOVA with Dunnett’s T3 test). (**b**) Representative immunoblots of liver lysates showing PTEN, p-AKT(Ser473), and pan-AKT; total protein staining used for normalization (endogenous PTEN = mPTEN; exogenous PTEN = *hPTEN*). (**c**) Quantification of PTEN protein levels across groups (Student’s *t*-test for wild-type (n = 4) vs. *Pten*^Δ5/+^ PBS (*n* = 8); Welch’s ANOVA for *Pten*^Δ5/+^-treated groups; 5E11 vg/mouse (*n* = 6), 2E12 vg/mouse (*n* = 9)). (**d**) Quantification of relative p-AKT(Ser473) levels normalized to pan-AKT (Welch’s *t*-test for wild-type (*n* = 8) vs. *Pten*^Δ5/+^ PBS (*n* = 12); Welch’s ANOVA with Dunnett’s T3 test for *Pten*^Δ5/+^-treated groups). Bar graphs display mean ± SD unless otherwise indicated. Individual data points (circles) represent individual mice. Significance levels: * *p* < 0.05, ** *p* < 0.01, *** *p* < 0.001, **** *p* < 0.0001.

Prior to evaluating therapeutic efficacy in the *Pten*^Δ5/+^ model, AAV9-mediated PTEN expression was validated in wild-type mice. These studies confirmed successful transgene expression and appropriate cellular and subcellular localization of PTEN by immunofluorescence (Figures S3 and S4). Following intravenous administration in *Pten*^Δ5/+^ mice, vector biodistribution analysis by qPCR demonstrated a strong dose-dependent accumulation of vector genomes in the liver eight months after treatment (Figure 3a). Consistent with this finding, hepatic PTEN protein expression was significantly increased in vector-treated *Pten*^Δ5/+^ mice compared with PBS-treated controls (Figure 3b,c). Restoration of PTEN expression was accompanied by a significant reduction in the elevated p-AKT/pan-AKT signalling observed in PBS-treated *Pten*^Δ5/+^ mice (*p* < 0.01; Figure 3b,d), indicating effective modulation of aberrant PI3K/AKT pathway activity.

### 3.4. Neonatal Intracranial scAAV9/Cβh-hPTEN Delivery Prevents Macrocephaly and Cortical Overgrowth in Pten^Δ5/+^ Mice

Neurological manifestations are well documented in PHTS, including macrocephaly, seizures, developmental delay, and ASD. Mouse models with germline or conditional *Pten* deletion replicate similar phenotypes, including brain overgrowth, behavioural abnormalities, and excitatory–inhibitory imbalance [7,9,33,34]. These models consistently exhibit increased absolute and relative brain mass in adulthood, with regional enlargement notably affecting the cortex and cerebellum. Our *Pten*^Δ5/+^ cohort demonstrated comparable structural brain abnormalities, which were not corrected by systemic scAAV9/Cβh-*hPTEN* administration (Figure S5), likely due to insufficient brain transduction. Consequently, we investigated the effects of direct intracranial vector delivery in female mouse neonates at two different doses (low: 5E10 and high: 1E11 vg/mouse) to assess its impact on brain development.

**Figure 4:**
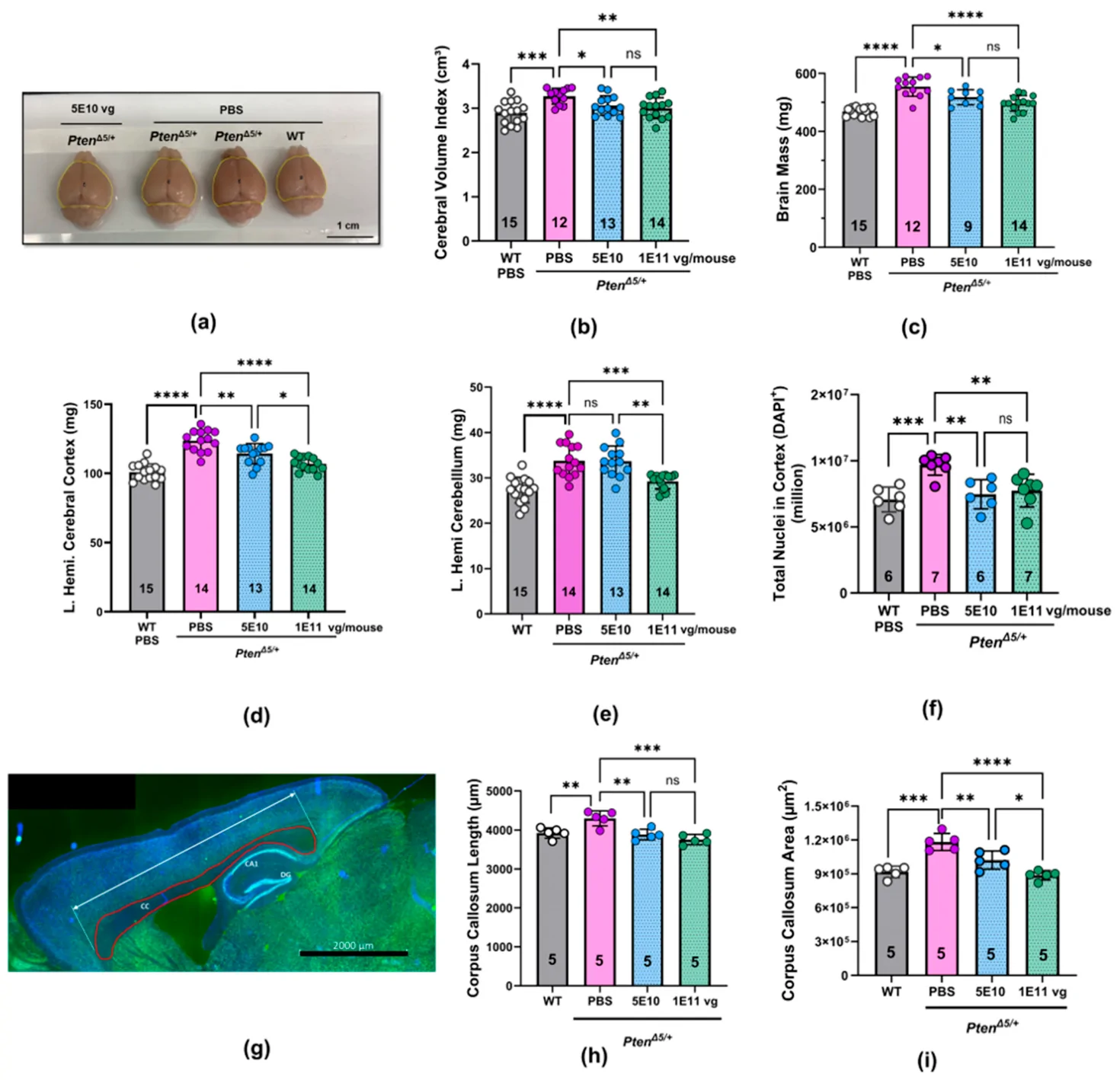
Intracranial scAAV9/Cβh-*hPTEN* restores altered neuroanatomical structures in *Pten*^Δ5/+^ female mouse: (**a**) Representative brain images taken 15 weeks after intracranial injection of either PBS (control) or scAAV9/Cβh-*hPTEN* into wild-type and *Pten*^Δ5/+^ female mouse neonates. Yellow outlines indicate the projected cerebral area used for ImageJ analysis. (**b**) The cerebral volume index calculated using ImageJ by multiplying the measured cerebral area by its corresponding perimeter (area × perimeter; cm^2^ × cm = cm^3^). This index was used as a surrogate measure for relative comparisons of cerebral size between experimental groups. (**c**) Absolute brain mass (mg) comparison across treatment groups. (**d**,**e**) Post-fixed left hemispheres were dissected to isolate and weigh the cerebral cortex (**d**) and cerebellum (**e**). (**f**) Quantification of total cortical nuclei using the isotropic fractionator method. (**g**) Representative micrograph of the right hemisphere stained with NeuroTrace™ 500/525 and DAPI. White arrow shows corpus callosum length; red outline delineates the corpus callosum area (scale bar: 2000 µm). (**h**,**i**) Quantification of corpus callosum length (**g**) and area (**i**) across experimental groups. Bar graphs show mean ± SD with individual animal data points (circles). Sample sizes are indicated within each bar. Statistical analysis: Two-tailed Student’s *t*-test (PBS-treated genotype comparison); one-way ANOVA with Tukey’s multiple comparisons test (among *Pten*^Δ5/+^-treated group). * *p* < 0.05, ** *p* < 0.01, *** *p* < 0.001, **** *p* < 0.0001, ns = not significant.

Experimental mice were euthanized at 15 weeks post-treatment by transcardial perfusion with PBS followed by 4% paraformaldehyde (PFA) to assess gross and microscopic brain structure. After fixation, two-dimensional (2D) images of the whole brains were acquired, and the projected cerebral areas were delineated using ImageJ version 1.54 (Figure 4a). Quantitative analysis revealed a significant 13% increase in cerebral volume index in PBS-treated *Pten*^Δ5/+^ mice relative to PBS-treated wild-type controls (t (25) = 4.296, *p* = 0.0002, mean ± SD; *Pten*^Δ5/+^ *n* = 12, 3.273 ± 0.177 cm^3^; wild-type *n* = 15, 2.896 ± 0.259 cm^3^). Intracranial administration of scAAV9/Cβh-*hPTEN* at either 5E10 or 1E11 vg/mouse resulted in a significant reduction in cerebral volume index in *Pten*^Δ5/+^ compared to PBS-treated *Pten*^Δ5/+^ mice (F (2,36) = 5.554, *p* = 0.0079; 5E10 vg/mouse *n* = 13, 3.063 ± 0.022 cm^3^; 1E11 vg/mouse *n* = 14, 3.003 ± 0.235 cm^3^; Figure 4b).

Additionally, we found a significant (~18%) increase in brain weight in PBS-treated *Pten*^Δ5/+^ mice compared to wild-type controls (t (14.35) = 8.318; mean ± SD, *Pten*^Δ5/+^ *n* = 12, 554.2 ± 32.9 (mg); wild-type *n* = 15, 469.1 ± 14.4 (mg), *p* < 0.0001), which was significantly (*p* < 0.0001) reduced following intracranial scAAV9/Cβh-*hPTEN* delivery (F (2,32) = 12.84; *Pten*^Δ5/+^ = PBS *n* = 12, 554.2 ± 32.9 (mg); 5E10 vg/mouse *n* = 9, 517.9 ± 26.1 (mg); 1E11vg/mouse *n* = 14, 496.6 ± 26.5 (mg), Figure 4c).

Previous studies have demonstrated widespread brain overgrowth affecting multiple regions in both humans and *Pten* haploinsufficiency mouse models [35,36]. To delineate regional effects and assess scAAV9/Cβh-*hPTEN* efficacy, we dissected the left hemisphere into cortex and cerebellum, weighing each separately. PBS-treated *Pten*^Δ5/+^ mice showed significantly increased weights of the cerebral cortex (t (27) = 8.875, *p* < 0.0001) and cerebellum (t (27) = 5.544, *p* < 0.0001) versus PBS-treated wild-type controls. Intracranial scAAV9/Cβh-*hPTEN* treatment resulted in a significant, dose-dependent reduction in mass of both regions (cortex: F (2,38) = 21.01, *p* < 0.0001; cerebellum: F (2,38) = 10.75, *p* = 0.0002; Figure 4d,e) of the *Pten*^Δ5/+^ treated mice.

To determine whether the increased cortical weight in *Pten*^Δ5/+^ mice was due to hyperplasia or hypertrophy, we used the isotropic fractionator method to quantify total cortical cell numbers. Consistent with previous findings [37], *Pten*^Δ5/+^ mice exhibited a significant increase in cortical cell number compared to wild-type controls (t (11) = 5.493, *p* = 0.0002). This elevation was significantly reduced by intracranial scAAV9/Cβh-*hPTEN* treatment (F (2,17) = 8.885, *p* = 0.0023), independent of dose (Figure 4f). Cortical cell density (cells/mg) remained unchanged between groups, indicating that cortical overgrowth was driven by increased cell number (hyperplasia) rather than increased cell size (Figure S6A).

Adult *Pten*^+/−^ mouse models exhibit increased total cell number, predominantly due to glial proliferation, alongside abnormal scaling of white matter regions including marked corpus callosum thickening [35,37]. To assess structural alterations in our *Pten*^Δ5/+^ cohort, we quantified corpus callosum length and area on immuno-stained midsagittal sections taken from the right hemisphere. Both dimensions were significantly increased in *Pten*^Δ5/+^ versus wild-type controls (length: t (8) = 3.575, *p* = 0.0072; area: t (11) = 6.468, *p* = 0.0002). Notably, intracranial scAAV9/Cβh-*hPTEN* administration normalized corpus callosum morphology in a dose-dependent manner, reflected by significant reductions in both length (F (2,12) = 16.60, *p* = 0.0003) and area (F (2,12) = 23.50, *p* < 0.0001) (Figure 4h,i).

**Figure 5:**
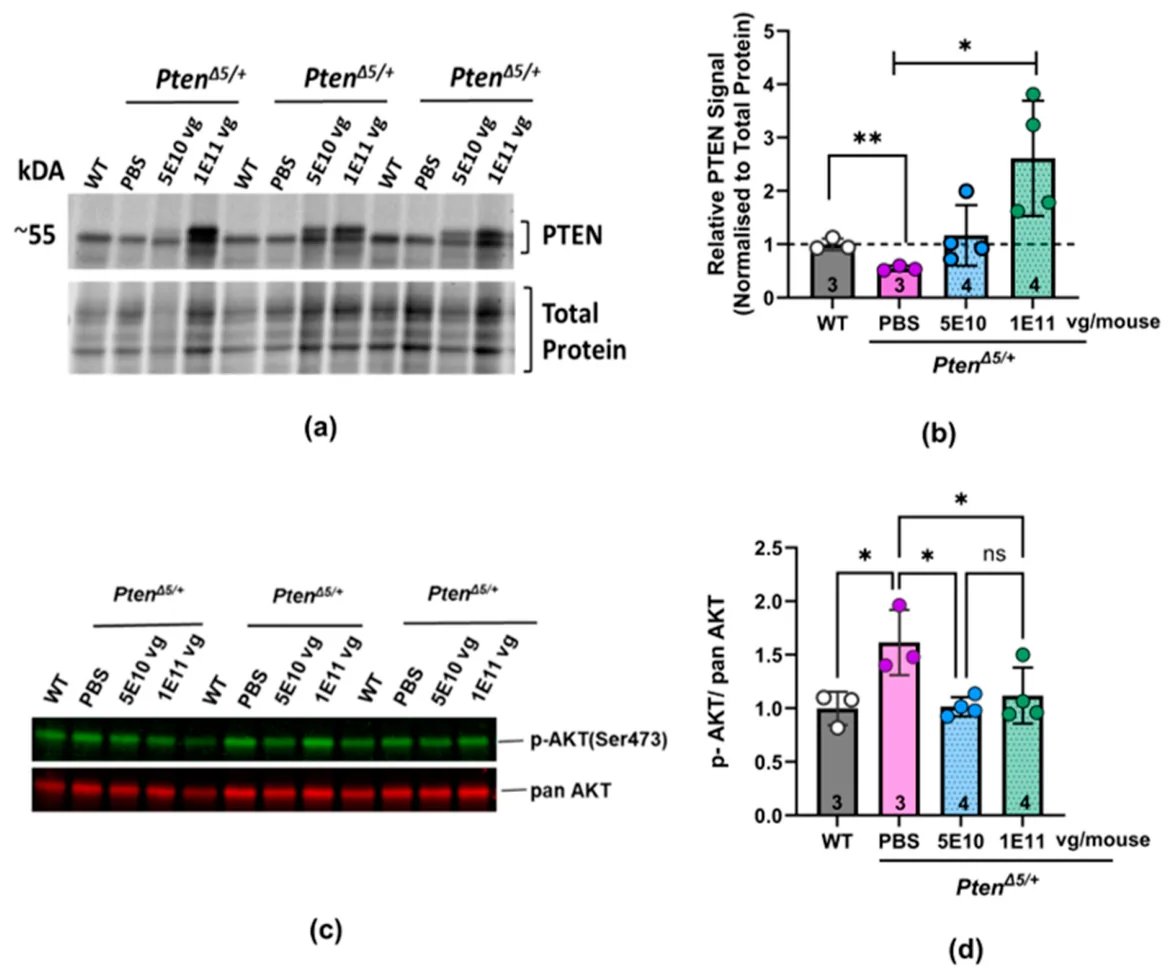
scAAV9/Cβh-*hPTEN* biodistribution restores PTEN expression and modulates cortical signalling in *Pten*^Δ5/+^ mice. (**a**) Representative immunoblots of cortex lysates from wild-type and *Pten*^Δ5/+^ mice labelled with anti-PTEN; total protein staining served as a loading control. (**b**) Quantification of relative PTEN expression in cortex lysates of treatment groups (Student’s *t*-test between genotype with PBS-treated group; Welch’s ANOVA among *Pten*^Δ5/+^-treated group). (**c**) Representative immunoblots for p-AKT Ser473 and pan-AKT in cortex lysates from wild-type and *Pten*^Δ5/+^ mice. (**d**) Quantification of relative p-AKT Ser473 levels normalized to pan-AKT in cortex lysates of treatment groups (Student’s *t*-test for PBS-treated genotypes; Welch’s ANOVA among *Pten*^Δ5/+^-treated group). All data are shown as mean ± SD with individual data points (* *p* < 0.05, ** *p* < 0.01, ns = not significant). Each data point shown in the graphs represents protein extracted from an individual mouse tissue sample, with each biological replicate corresponding to a different animal in the respective experimental group.

Following administration of either 5E10 or 1E11 vg/mouse, biodistribution analysis revealed a significant increase in vector copies in the cortex (mean ± SD; 2.82 ± 1.5 *hPTEN* copies/diploid genome for 5E10 vg/mouse; 3.79 ± 1.3 *hPTEN* copies/diploid genome for 1E11 vg/mouse; *p* = 0.002) and cerebellum (mean ± SD; 0.52 ± 0.2 *hPTEN* copies/diploid genome for 5E10 vg/mouse; 0.99 ± 0.3 *hPTEN* copies/diploid genome for 1E11 vg/mouse, *p* = 0.0013) of the vector-treated *Pten*^Δ5/+^ treated mice compared to the PBS-treated control (Figure S6B,C). Western blot analysis of the cortical tissues revealed that baseline PTEN expression in PBS-treated *Pten*^Δ5/+^ mice was reduced to 55% of wild-type levels. In contrast, *Pten*^Δ5/+^ mice treated with the low dose exhibited cortical PTEN levels comparable to wild-type, whereas those that received the high dose showed a significant increase (~3.4 fold, *p* < 0.05) in cortical PTEN expression compared to the PBS-treated *Pten*^Δ5/+^ (Figure 5a,b). Additionally, p-AKT (Ser473) signalling, assessed as the ratio of phosphorylated to total AKT protein, was significantly reduced in *Pten*^Δ5/+^ mice treated with either vector dose compared with PBS-treated controls, returning to levels comparable with wild-type (Figure 5c,d).

PTEN and mTORC1 signalling pathways play a critical role in regulating normal social behaviour with evidence of altered social behaviours following either PTEN overexpression or mTOR signalling pathway inhibition in wild-type mice [38,39,40,41]. Therefore, we conducted social preference tests in the treated groups to investigate potential PTEN overexpression-related behaviour deficits. Consistent with our previous findings (Figure S1K), no significant difference in social preference was observed between PBS-treated wild-type and *Pten*^Δ5/+^ mice (t (27) = 1.050, *p* = 0.3029; Figure S6D). Importantly, intracranial administration of scAAV9/Cβh-*hPTEN* at both tested doses did not alter social behaviour in *Pten*^Δ5/+^ mice (H (2) = 0.7062, *p* = 0.7020; Figure S6D). This finding is particularly relevant in light of the observed PTEN overexpression at the higher vector dose, supporting the safety and tolerability of the gene therapy and indicating no adverse effects on social interaction in this model of *Pten* haploinsufficiency.

## 4. Discussion

Given the high penetrance of PHTS and the associated resistance to chemotherapy, immunotherapy, and radiotherapy [42,43,44], treating the root cause of the disease using gene therapy-based approaches have the potential to offer significant clinical advantages. This study provides proof-of-concept evidence that AAV9-mediated delivery of a codon-optimized human *PTEN* transgene can partially rescue disease phenotypes in a PHTS mouse model, while also revealing key limitations that may hinder clinical translation.

### 4.1. Systemic scAAV9/Cβh-hPTEN Fails to Suppress Lymphoid Tumourigenesis but Prevents PTEN-Haploinsufficiency-Associated Hepatic Steatosis

Systemic administration of the scAAV9/Cβh-*hPTEN* vector did not delay tumour onset or reduce tumour growth in *Pten*^Δ5/+^ mice, even at the highest doses tested. This result should be interpreted in the context of the broader phenotype of *Pten*^Δ5/+^ mice, which recapitulate hallmark features of *PTEN* Hamartoma Tumour Syndrome (PHTS), including spontaneous tumorigenesis with lymph node hyperplasia as the predominant tumour phenotype [16,17,18,19]. Analysis of postmortem tissues showed that neither vector genomes nor vector-derived PTEN expression was detectable in the lymph nodes 8 months after treatment, suggesting either inefficient initial transduction and/or loss of vector genomes or transduced cells. These findings may reflect subtherapeutic vector accumulation in lymphoid cells or elimination via immune-mediated silencing and high lymphocyte turnover [45,46], both of which are known to restrict stable transgene expression and thereby limit the efficacy of AAV-based gene therapy. As vector biodistribution and transgene expression were assessed only at the terminal study endpoint, the present data cannot distinguish between limited initial lymphoid transduction and subsequent vector clearance or loss of transduced lymphoid cells. Future studies incorporating early post-injection biodistribution analyses and longitudinal assessment of vector persistence will be important to define the mechanisms underlying the limited efficacy observed in lymph nodes. Elevated p-AKT (Ser473) levels, indicative of persistent PI3K/AKT/mTOR pathway activation and ongoing tumour progression, were maintained in lymph nodes of vector-treated *Pten*^Δ5/+^ mice, further confirming inadequate pathway suppression [47,48].

Although systemic scAAV9/Cβh-*hPTEN* administration had little impact on lymph node tumorigenesis and failed to delay tumour onset or reduce lymphoid tumour growth, it produced a clear therapeutic benefit in the liver, where it reduced the incidence of steatosis and inflammatory foci and preserved hepatic architecture in *Pten*^Δ5/+^ mice. These focal fatty lesions were identified as focal steatotic changes by H&E morphology and Oil Red O staining; however, additional histopathological and molecular analyses would be required to determine whether they are purely metabolic or potentially pre-neoplastic. This therapeutic effect, mediated by vector-derived PTEN, probably prevented progression toward advanced-stage disease, as previous reports have shown that uncorrected PTEN loss in hepatocytes leads to dysregulated lipid metabolism, chronic inflammation, and subsequent progression to hepatocellular carcinoma (HCC) [49]. In the current study, restoring PTEN expression likely reactivated its key tumour-suppressive functions—including inhibition of the PI3K–AKT–mTOR signalling cascade, restoration of lipid homeostasis, and attenuation of hepatocyte proliferation—all of which are fundamental to preventing neoplastic transformation [50]. The observed reduction in hepatic steatosis and inflammatory foci suggests that sustained PTEN expression dampened aberrant Akt-driven lipid accumulation and reduced inflammatory cell infiltration, processes known to accelerate the transition from steatosis to non-alcoholic steatohepatitis (NASH) and HCC [51,52]. This is consistent with evidence that PTEN deficiency promotes hepatocyte hypertrophy, DNA damage, and metabolic stress through unchecked PI3K–mTOR activation [53]. Restoration of PTEN in our model therefore likely reinstated negative feedback on this pathway, resulting in reduced oxidative stress and maintenance of genomic stability in hepatocytes. Importantly, these findings resonate with emerging translational evidence that PTEN restoration—either via canonical PTEN or the secreted PTEN-Long isoform [54]—can suppress proliferation and migration of hepatic tumour cells, enhance apoptosis, and induce autophagy [50]. In this context, the dose-dependent restoration of hepatic PTEN expression observed here not only suggests effective vector transduction but also supports PTEN’s pleiotropic function in maintaining hepatic integrity and immune equilibrium. Additionally, restoring the PTEN level can interrupt pathways leading to hepatic oncogenesis positioning *PTEN* gene therapy as a potential strategy to prevent liver malignancy in PTEN-deficient states and, potentially, to extend its application to broader metabolic liver disorders with oncogenic risk.

### 4.2. Intracerebroventricular PTEN Gene Therapy Normalizes Brain Overgrowth and Cellularity in Neonatal Pten^Δ5/+^ Mice

In addition to its marked benefit on hepatic pathology, scAAV9/Cβh-*hPTEN* gene therapy also demonstrated significant efficacy in the central nervous system. In this study, neonatal intracerebroventricular (ICV) [55,56] delivery of scAAV9/Cβh-*hPTEN* demonstrated marked therapeutic potential in mitigating the macrocephalic phenotype characteristic of *Pten*^Δ5/+^ mice. Restoration of brain size and total cell count following AAV9-mediated PTEN expression underscores PTEN’s pivotal role in regulating neuronal and glial proliferation via the PI3K–AKT–mTOR signalling cascade, a pathway whose dysregulation underlies both structural and functional abnormalities in *PTEN* haploinsufficiency syndromes [37]. Restoration of PTEN expression through AAV-mediated delivery suggests a targeted means of correcting these molecular imbalances by reinstating negative feedback control on AKT/mTOR-dependent protein synthesis and cell growth [57].

Macrocephaly is a hallmark feature across the clinical spectrum of *PTEN* Hamartoma Tumour Syndrome (PHTS), including Cowden syndrome, Bannayan–Riley–Ruvalcaba syndrome, and *PTEN*-related autism spectrum disorder (ASD). Defined as an occipitofrontal circumference (OFC) greater than two standard deviations above the mean, macrocephaly is present in approximately 90–95% of individuals with pathogenic germline *PTEN* variants, making it one of the most consistent clinical manifestations of PHTS [5,6,33,58,59]. In addition to somatic overgrowth, PTEN plays a critical role in brain development and neurodevelopmental function. Germline *PTEN* pathogenic variants have been identified in approximately 10–20% of individuals with ASD and macrocephaly, highlighting a well-established association between PTEN dysfunction, abnormal brain growth, and neurodevelopmental disorders [5,6,59,60]. Furthermore, intracranial neoplasms have been reported in approximately 10% of individuals with PHTS [61,62], further underscoring the importance of PTEN in regulating both neurodevelopment and tumour suppression.

The phenotypic normalization of brain size and cortical architecture aligns with prior pharmacological studies demonstrating partial rescue of overgrowth by mTOR inhibition, yet gene therapy achieves a more direct correction of the underlying genetic deficiency. In contrast to systemic rapamycin, which transiently suppresses mTOR activity and carries off-target metabolic consequences [63], PTEN restoration is expected to stabilize signalling homeostasis while preserving physiological mTOR functions required for neuronal maturation. Mechanistically, this approach targets the root cause of aberrant cellular expansion by restoring both the lipid and protein phosphatase activities of PTEN, which govern neuronal size, dendritic architecture, and cortical organization [64].

Normalization of total cortical cell number following PTEN replacement suggests suppression of hyperplastic processes rather than compensatory changes in cell density, consistent with evidence that PTEN restrains gliogenesis and promotes balanced neuron-glia ratios during cortical development [65]. The observed reduction in p-AKT levels to near wild-type values further confirm effective reactivation of PTEN functionality at critical signalling nodes. Importantly, these corrections occurred without the behavioural toxicity previously reported in PTEN-overexpressing models, which have been associated with increased depression-like behaviours, reduced social interactions [40], or, in some cases, enhanced social behaviour [41]. The absence of social deficits or adverse behavioural responses further reinforces the therapeutic potential of the vector system, which achieved molecular correction without triggering PTEN overexpression-related impairments in neuronal activity or affective regulation [38,39,40,41]. Nevertheless, while assessments of general health, body weight, and behaviour revealed no overt toxicity, a comprehensive evaluation of the safety of PTEN overexpression—particularly following high-dose ICV administration—will require dedicated studies incorporating neurological, biochemical, immunological, and histopathological endpoints.

It is important to note that although intracerebroventricular PTEN gene therapy corrected several neuroanatomical abnormalities and restored PI3K/AKT signalling, no robust behavioural deficits were detected in *Pten*^Δ5/+^ mice under the conditions examined. Consequently, the present study could not determine whether these structural improvements translate into functional neurological benefits. While the observed anatomical rescue demonstrates biological efficacy within the central nervous system, it should not be interpreted as evidence of improved cognitive, behavioural, or other neurodevelopmental outcomes. Future studies using models with well-defined neurobehavioral phenotypes and incorporating additional functional assessments, including electrophysiological analyses and seizure susceptibility testing, will be required to establish the therapeutic potential of *PTEN* gene therapy for the neurodevelopmental manifestations of PHTS.

## 5. Conclusions

Overall, these findings demonstrate that effective restoration of PTEN expression by both systemic and intracranial AAV9-mediated gene therapy has the potential to correct the underlying PI3K–AKT–mTOR dysregulation and thereby ameliorate key molecular and phenotypic abnormalities associated with the neurodevelopmental and hepatic manifestations of *PTEN* Hamartoma Tumour Syndrome (PHTS). In the liver, systemic scAAV9/Cβh-*hPTEN* reduced early steatosis and inflammatory lesions, preserving hepatic architecture and interrupting pathways that drive progression toward hepatocellular carcinoma. Additionally, in the CNS, neonatal intracerebroventricular (ICV) injection produced robust brain-specific transduction and reversed macrocephaly and cortical hypercellularity, supporting the development of CNS-directed *PTEN* gene therapy as a preventive and disease-modifying strategy not only for PHTS but also for neurodevelopmental disorders characterized by dysregulated PI3K–AKT–mTOR activity.

Nonetheless, several important limitations remain. PHTS represents a complex multisystem syndromic disorder, and achieving therapeutically meaningful PTEN restoration across all clinically relevant tissues remains inherently challenging. In particular, the relatively low transduction efficiency observed in lymphoid and certain peripheral tissues, alongside the technical and developmental constraints of early-postnatal ICV delivery, underscores the need for next-generation vectors with improved and broader tissue tropism, including engineered or hybrid AAV variants capable of more precise and balanced biodistribution across immune, epithelial, and neural compartments [66]. Moreover, the long-term safety of sustained PTEN expression—especially within neuronal circuits highly sensitive to perturbations in PI3K/AKT/mTOR signalling—requires careful evaluation.

Another limitation of this study is the use of a C-terminal FLAG tag to distinguish vector-derived PTEN from endogenous PTEN. Although FLAG-tagged PTEN exhibited appropriate cytoplasmic and nuclear localization and effectively suppressed p-AKT signalling in vivo, the possibility that the tag alters PTEN stability, PDZ-dependent protein interactions, or other non-canonical functions cannot be excluded. Future studies directly comparing tagged and untagged PTEN constructs will be important to fully assess any functional consequences of C-terminal tagging.

An additional limitation of this study is the absence of an empty AAV9 control vector and a wild-type + AAV9/Cβh-*hPTEN* treatment group. Although PBS-treated animals served as disease controls, these additional control groups would facilitate discrimination between vector-related and PTEN-mediated effects and would further inform the safety profile of PTEN overexpression in normal tissues, particularly following intracerebroventricular administration.

Finally, the therapeutic efficacy studies were conducted predominantly in female *Pten*^Δ5/+^ mice, which were selected because of their earlier onset and more aggressive disease phenotype. While this study design facilitated the evaluation of treatment efficacy, it limits assessment of potential sex-specific differences in therapeutic response. Given that PTEN-associated phenotypes and disease progression may differ between males and females, the current findings should not be assumed to be fully generalizable across sexes. Future studies directly comparing male and female mice will be important to determine whether the efficacy and safety of *PTEN* gene therapy are influenced by biological sex.

Achieving optimal targeting across all relevant tissues therefore remains challenging, and combining *PTEN* gene therapy with pharmacological pathway modulators, such as PI3K or mTOR inhibitors, may further enhance therapeutic precision and reduce compensatory signalling. Emerging strategies, including CRISPR-based PTEN correction and delivery of the secreted PTEN-Long isoform, also represent viable directions for broadening treatment scope and extending efficacy to non-transduced cells [50,67]. Collectively, these findings support the further development of durable, tissue-optimized PTEN restoration strategies aimed at preventing disease-associated pathology and re-establishing homeostatic control in PTEN-deficient disorders. However, the present findings should be considered proof of concept, particularly for CNS-directed applications. Although AAV-mediated PTEN gene replacement successfully corrected PTEN-dependent molecular and neuroanatomical abnormalities in vivo, important translational challenges remain, including dose scaling, optimization of the therapeutic window, efficient targeting of clinically relevant tissues, pre-existing anti-AAV immunity, vector immunogenicity, and the long-term consequences of sustained PTEN expression. Furthermore, neonatal intracerebroventricular delivery in mice does not directly model clinical administration in patients. Addressing these challenges through improved vector engineering, clinically relevant delivery approaches, and comprehensive long-term safety studies will be essential before *PTEN* gene therapy can be translated to the clinic.

## Data Availability

The data supporting the findings of this study are included within the article and its Supplementary Materials. Additional datasets used or analyzed during this study are available from the corresponding author upon request.

## Supplementary Materials

The following supporting information can be downloaded at: https://www.mdpi.com/article/10.3390/pharmaceutics18080922/s1, Figure S1: Female *Pten*^Δ5/+^ mice exhibit earlier tumour onset than males, while both sexes display increased brain mass; Figure S2: Predominant tumour development in the lymph nodes with limited vector biodistribution; Figure S3: Validation of AAV9-mediated PTEN expression in the liver of wild-type mice; Figure S4: Validation of AAV9-mediated PTEN expression and subcellular localization in the liver of wild-type mice; Figure S5: Lack of rescue of macrocephaly and no body weight changes in *Pten*^Δ5/+^ mice following intravenous delivery of scAAV9/Cβh-*hPTEN*; Figure S6: scAAV9/Cβh-*hPTEN* biodistribution in the brain of vector-treated *Pten*^Δ5/+^ mice.

## Abbreviations

The following abbreviations are used in this manuscript:

PHTS	*PTEN* Hamartoma Tumour Syndrome
*hPTEN*	Human phosphatase and tensin homologue
PI3K	Phosphatidylinositol 3-kinases
AKT	Protein kinase B
scAAV	Self-complementary adeno-associated virus
AAV9	Adeno-associated virus serotype 9
CβA	Chicken beta-actin
Cβh	Hybrid form of CBA
H&E	Haematoxylin and eosin
IV	Intravenous
ICV	Intracerebroventricular
vg	Vector genome
FFPE	Formalin-fixed and paraffin-embedded
PBS	Phosphate-buffered saline
