# Supplementary material for: AAV9-Mediated PTEN Gene Therapy Rescues Hepatic Pathology and Neuroanatomical Abnormalities in a Murine Model of PTEN Hamartoma Tumour Syndrome
Source: Pharmaceutics. 2026 Jul 27;18(8):922. doi: 10.3390/pharmaceutics18080922 (PMC13516674; doi:10.3390/pharmaceutics18080922)
Supplement: Supplementary file 1 [file pharmaceutics-18-00922-s001.zip › pharmaceutics-4387556-supplementary.pdf]

Supplementary Materials

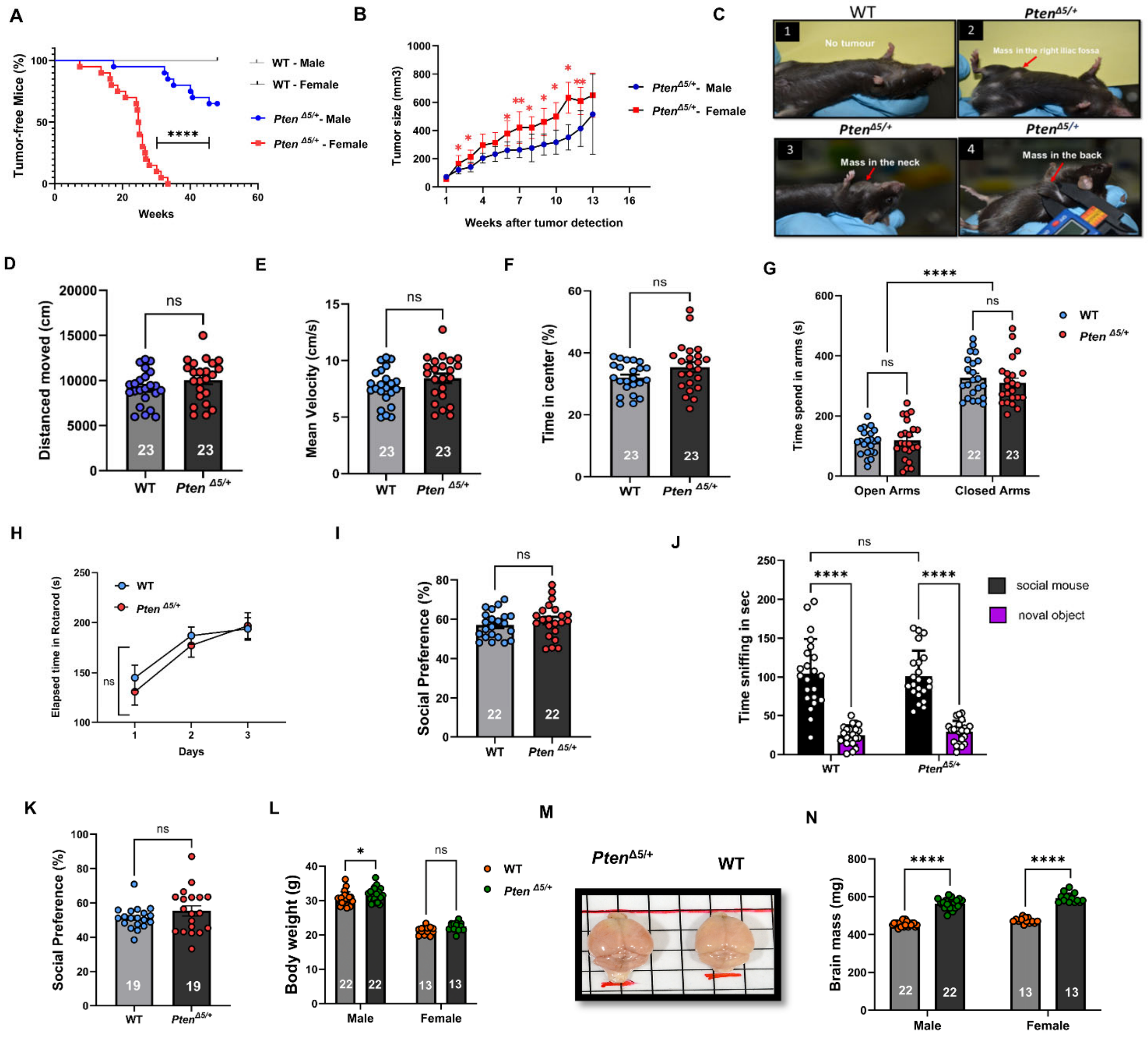

Figure S1. Female $\textbf{\textit{Pten}}^{\Delta5/+}$ **mice exhibit earlier tumour onset than males, while both sexes display increased brain mass**: **(A)** Kaplan–Meier survival curves illustrating significantly increased tumour incidence and earlier tumour development in female $Pten^{\Delta5/+}$ mice relative to wild-type (WT) controls (Log-rank (Mantel-Cox) test; **(B)** Tumour growth kinetics in $Pten^{\Delta5/+}$ female and male mice were assessed by weekly digital calliper measurements. Total tumour burden was defined as the sum of the volumes of all tumours present in each mouse. Mice were euthanised when the cumulative tumour volume reached 1.5 cm^3, in accordance with humane endpoint criteria. Data were analysed using a Mixed-effects model with Dunnett's post hoc test; **(C)** Representative micrographs showing wild-type mice (1) without tumours and visible tumours in $Pten^{\Delta5/+}$ mice (2–4); **(D)** Bar plot illustrating mean total distance travelled during a 20-minute session in the open field arena at 8 weeks postnatally of $Pten^{\Delta5/+}$ and wild-type male mice; **(E)** A similar bar plot showing the average velocity and; **(F)** Motion time spent in the centre area as a percentage of total time in the arena **(G)** Plot showing the movement time of ($Pten^{\Delta5/+}$ and wild-type male) mice spent in the open arms or closed arms of the elevated plus maze platform; **(H)** The graph displays the mean duration for each 10-week-old $Pten^{\Delta5/+}$ and wild-type male to successfully complete the Rotarod test (by not falling or passively rotating on the rod), across three sets for each day**.;** **(I)** The plot depicts the percentage of social preference in 12-week-old $Pten^{\Delta5/+}$ and wild-type male, which is calculated as the time spent with a novel mouse divided by the total time spent in chambers with both the novel mouse and a novel object, multiplied by 100 ; **(J)** The graph shows the time spent sniffing the novel mouse versus the novel object in the $Pten^{\Delta5/+}$ and wild-type male mice; **(K)** The plot illustrates the percentage of social preference by three chamber social test in 12-week old $Pten^{\Delta5/+}$ and wild-type female; **(L)** Body weights at 15 weeks show no significant differences between $Pten^{\Delta5/+}$ and wild-type females; **(M)** Brain images

showing enlarged enlargement *Pten*$^{\Delta5/+}$ mouse brains compared to wild-type controls; **(N)** Quantification of brain weights at 15 weeks demonstrates significant mass increase in *Pten*$^{\Delta5/+}$ mice in both sexes (Two-way ANOVA with Šídák's multiple comparisons). Data are presented as mean ± SD with individual data points shown as circles; sample sizes are indicated on bars. Statistical significance: *$p < 0.05$, **$p < 0.01$, ***$p < 0.001$, ****$p < 0.0001$; ns = not significant.

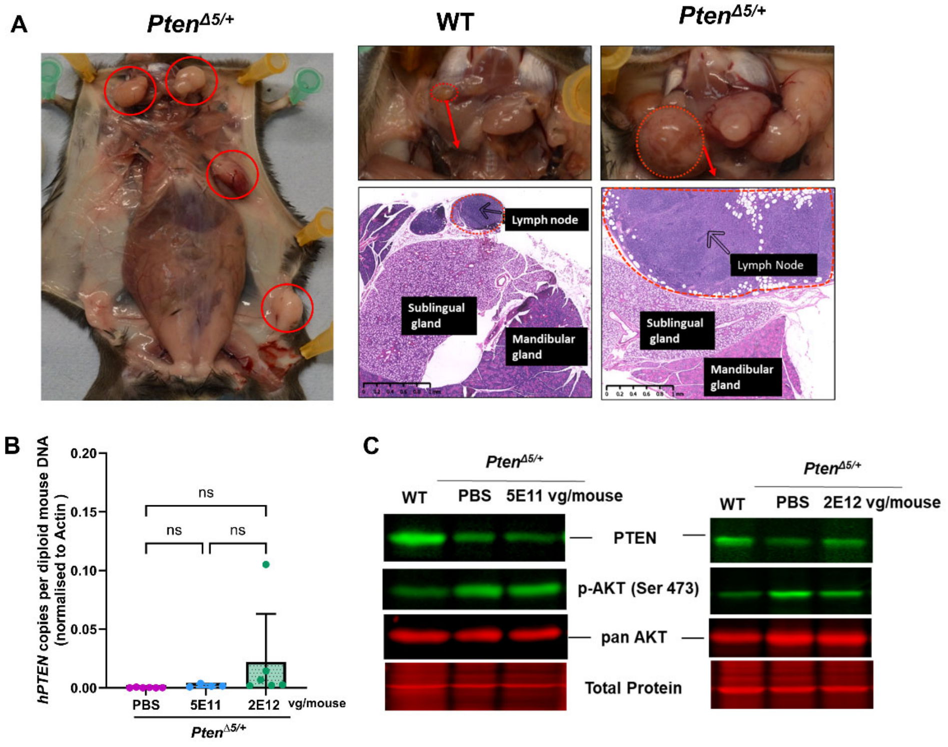

Figure S2: Predominant tumour development in the lymph nodes with limited vector biodistribution: (A) Left: anatomical locations of palpable tumour measurements following detection. Right: representative images of cervical lymph nodes (top; circled) in wild-type and in *Pten*$^{\Delta5/+}$ mice (note enlarged cervical lymph nodes in *Pten*$^{\Delta5/+}$ example). H&E-stained sections of lymph nodes and surrounding salivary glands (bottom); **(B)** Restricted scAAV9/Cβh-*hPTEN* vector biodistribution in cervical lymph nodes eight months post-injection; (Welch's ANOVA; *Pten*$^{\Delta5/+}$ PBS $n = 6$; 5E11vg/mouse $n = 4$; 2E12 vg/mouse $n = 6$); **(C)** Representative immunoblots for PTEN, p-AKT, pan-AKT, and total protein (for normalization) in lymph node lysates. Data are presented as mean ± SD with individual data points shown (circles). ns = not significant.

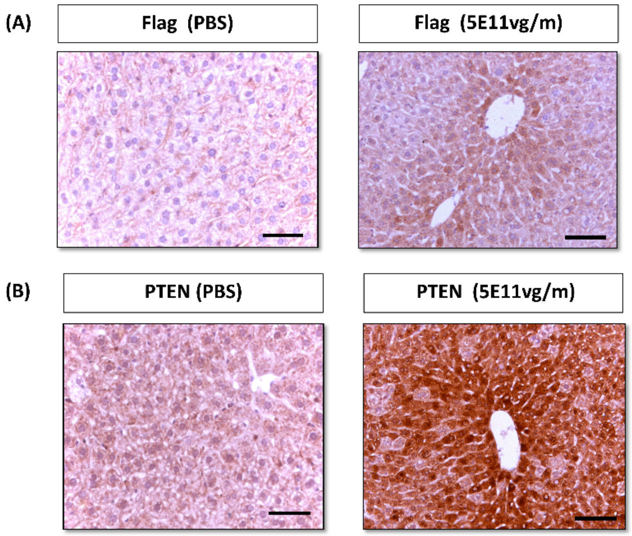

Figure S3: Validation of AAV9-mediated PTEN expression in the liver of wild-type mice: (A) Representative immunohistochemistry (IHC/DAB) images of liver sections (7 µm) stained with an anti-FLAG antibody from wild-type mice treated with PBS or scAAV9/Cβh-*hPTEN* (5 E11 vg/mouse). These experiments were performed as part of the vector validation studies to confirm successful AAV9-mediated expression of the FLAG-tagged human PTEN transgene prior to therapeutic evaluation in *Pten*$^{\Delta 5/+}$ mice. Brown DAB staining in scAAV9/Cβh-*hPTEN* -treated livers indicates expression of vector-derived human PTEN. **(B)** Representative IHC/DAB images of liver sections (7 µm) stained with an anti-PTEN antibody from wild-type mice treated with PBS or scAAV9/Cβh-*hPTEN* (5E11 vg/mouse). PBS-treated mice show endogenous murine PTEN expression, whereas scAAV9/Cβh-*hPTEN* -treated mice exhibit increased PTEN immunoreactivity resulting from the combined detection of endogenous mouse PTEN and vector-derived human PTEN.

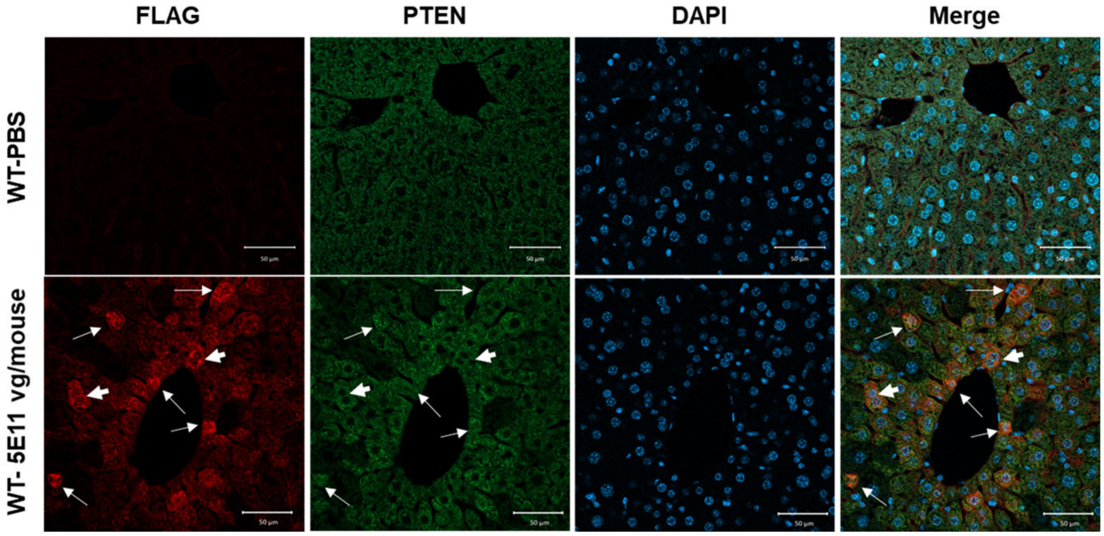

Figure S4: Validation of AAV9-mediated PTEN expression and subcellular localization in the liver of wild-type mice: Representative immunofluorescence images of liver sections from wild-type mice treated with PBS (top panel) or scAAV9/Cβh-*hPTEN* (5E11 vg/mouse) (bottom panel), stained with anti-FLAG (red), anti-PTEN (green), and DAPI (blue) to identify nuclei. These studies were performed as part of the vector validation experiments to confirm successful AAV9-mediated expression of human PTEN and to assess its cellular and subcellular localization prior to therapeutic evaluation in *Pten*$^{\Delta 5/+}$ mice. FLAG-positive cells in scAAV9/Cβh-*hPTEN*-treated livers confirm expression of vector-derived human PTEN. PTEN immunoreactivity was detected in both the cytoplasm and nucleus, consistent with the distribution of endogenous PTEN. Thin white arrows indicate PTEN localization in both cellular compartments, whereas thick arrowheads identify transduced hepatocytes exhibiting prominent expression of vector-derived PTEN. Scale bar = 50 µm.

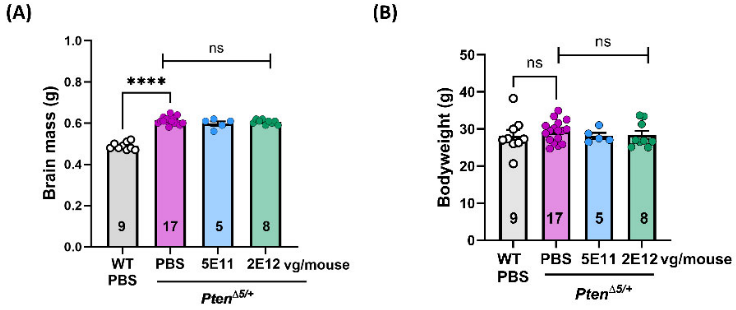

Figure S5: Lack of rescue of macrocephaly and no body weight changes in *Pten*$^{\Delta 5/+}$ mice following IV delivery of scAAV9/Cβh-*hPTEN*: (A) Bar graph comparing absolute brain weight across the experimental cohort at 8 months post-injection; **(B)** Final bodyweight of the experimental cohort. Individual data points (circles) represent single animals, and sample sizes are indicated within the bars. Data are presented as mean ± SD. Genotype comparison

(WT-PBS vs *Pten*$^{\Delta5/+}$ PBS) was performed with two-tailed Student's *t-test*, while the comparison among *Pten*$^{\Delta5/+}$ groups (PBS, 5E11 vg/mouse and 2E12 vg/mouse) was conducted with One-way ANOVA test. (****p < 0.0001, ns = not significant).

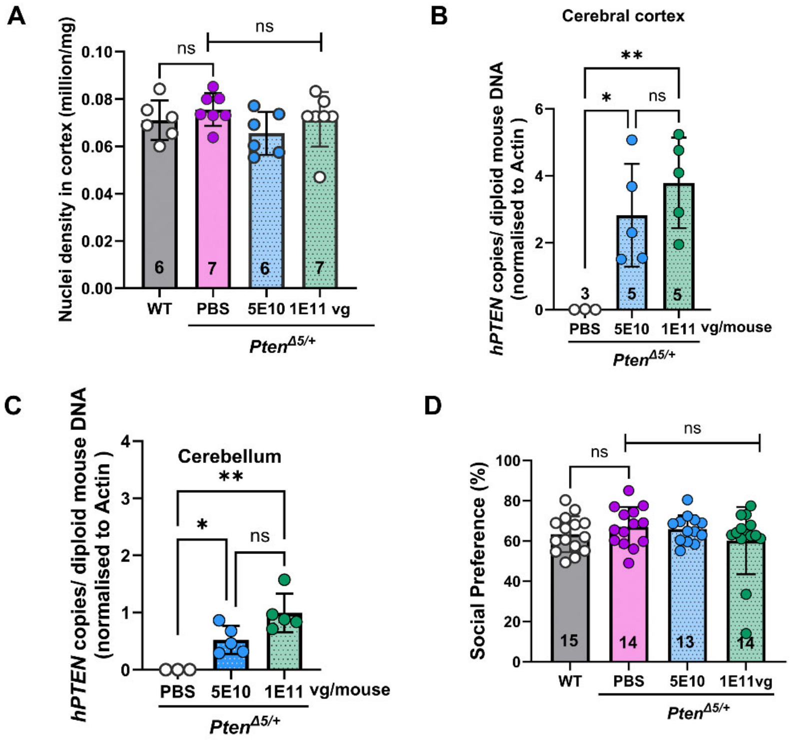

Figure S6: scAAV9/Cβh-*hPTEN* biodistribution in the brain of vector treated *Pten*$^{\Delta5/+}$ mice: (A) Graph demonstrates no significant change in nuclei density among treated groups. Nucleal density was calculated by dividing the total cell number by the mass in the cortex of the left hemisphere; **(B&C)** scAAV9/Cβh-*hPTEN* vector genome copies per diploid genome in the cerebral cortex and cerebellum at 15 weeks, quantified by qPCR. Sample sizes are indicated within or above each bar (Welch's ANOVA with Dunnett's T3 test); **(D)** Social preference in the three-chamber test, expressed as the percentage of time spent in the chamber with a social mouse versus a novel object (Student`s *t*-test between genotype with PBS treated; Kruskal–Wallis test among *Pten*$^{\Delta5/+}$ treated group). All Data are shown as mean ± SD with individual data points (*p < 0.05, **p < 0.01, ns = not significant).
